# Carbon Dioxide and Hemoglobin at Presentation with Hypertrophic Pyloric Stenosis—Are They Relevant? Cohort Study and Current Opinions

**DOI:** 10.3390/children12070885

**Published:** 2025-07-04

**Authors:** Ralf-Bodo Tröbs, Hiltrud Niggemann, Grigore Cernaianu, Andreas Lipphaus, Matthias Nissen

**Affiliations:** 1 Department of General-, Visceral- and Pediatric Surgery, St. Vinzenz Krankenhaus, 33098 Paderborn, Germany; 2 Division of Pediatric Surgery, University Hospital Cologne, Kerpenerstr. 62, 50937 Köln, Germany; niggemann@p-wert.de (H.N.); grigore.cernaianu@uk-koeln.de (G.C.); 3 Department of Pediatric Surgery, Ruhr-University of Bochum, St. Marie’s Hospital Witten, Sankt Elisabeth Gruppe Herne, 58452 Witten, Germany; andreas.lipphaus@rub.de (A.L.); matthias.nissen@elisabethgruppe.de (M.N.)

**Keywords:** infantile hypertrophic pyloric stenosis, carbon dioxide, hemoglobin, respiratory depression

## Abstract

**Highlights:**

**Background**

- In infantile hypertrophic pyloric stenosis, recurrent vomiting of gastric contents leads to metabolic alkalosis, which is compensated for by an increase in carbon dioxide.
- Carbon dioxide acts as a respiratory stimulus a priori, but at high concentrations it may have a depressant effect on respiration.
- The role of blood carbon dioxide tension (pCO_2_) in the development of respiratory problems remains unclear.

**Material and Methods**

- A retrospective monoinstitutional cohort study was conducted on 105 infants.
- The following parameters were recorded on admission: biometrics, acid/base status, hemoglobin, lactate and sodium.
- The present study aims to determine the interaction between hemoglobin and the other acid/base components on pCO_2_.

**Results**

- Hypercapnia was observed in a relevant number of children.
- Infants exhibiting hypercapnia have been observed to demonstrate signs of hemoconcentration.
- A positive correlation was identified between the concentration of hemoglobin and the partial pressure of carbon dioxide.
- The lactate level was not affected by the elevated partial pressure of carbon dioxide (pCO_2_).

**Conclusions**

- There is a dependence of pCO_2_ on hemoglobin.
- An increase in carbon dioxide cannot easily be attributed to reduced carbon dioxide transport function due to hemoglobin deficiency.
- The physico-chemical Stewart model is a theoretical framework that can be utilized to elucidate the complex pathophysiology of gastric alkalosis in IHPS. This approach offers a novel perspective on the investigation of this condition.

**Abstract:**

Background: Recurrent vomiting in infantile hypertrophic pyloric stenosis (IHPS) leads to metabolic alkalosis and a respiratory-driven compensatory hypercapnia. Alkalosis has been identified as the main causal factor for respiratory depression on admission. The value of contribution of hemoglobin and carbon dioxide partial pressure to this phenomenon will be evaluated. Materials and Methods: A retrospective cohort study was conducted on 105 infants with IHPS. The acid/base status, including levels of hemoglobin and lactate, were recorded. Statistical comparisons, correlation analysis, linear regression and multivariate regression analysis were applied. Results: Hypercapnia was associated with hemoconcentration. We found a positive correlation was found between pCO_2_ and hemoglobin (*p* = 0.042). The multivariate linear regression analysis showed that pCO_2_ is dependent on hemoglobin (*p* = 0.002). Lactate, which is used as a marker for anaerobic glycolysis, showed no systematic correlation with pCO_2_. Conclusions: An increase in carbon dioxide cannot easily be attributed to a reduced transport function of carbon dioxide due to hemoglobin deficiency. Further investigation is needed to determine the extent to which low hemoglobin levels and increased pCO_2_ interact with hemoconcentration to contribute to respiratory problems.

## 1. Introduction

Infantile hypertrophic pyloric stenosis (IHPS) is a common and enigmatic surgical disease of first-trimester infants with a peak incidence in the fifth week of life. Traditionally, the incidence of IHPS is estimated at around 1:300. A decreasing incidence is described in the United States and Germany [1,2,3]. Nevertheless, this trend could not be substantiated in the Netherlands. The incidence rate there was 1.28 per 1000 live births [4]. A particularly low incidence is also reported for Africa and Asia [5,6]. There is a clear male predominance with a gender ratio at 5 to 6:1. Clinically, affected babies with IHPS suffer from intractable propulsive and non-bilious, sometimes hematin-stained vomiting due to gastric outlet obstruction. This results in a loss of gastric fluids that mainly contain water, hydrochloric acid, and electrolytes. Repeated loss of hydrogen ions, chloride and sodium leads to an increased hypochloremic, hypokaliemic metabolic alkalosis [7,8].

Standard treatment for IHPS is surgical intervention (Fredet–Weber–Ramstedt’s operation). It is imperative that any fluid, electrolyte and acid/base imbalance must be corrected prior to the procedure.

In essence, infants with IHPS typically exhibit a favorable prognosis, characterized by a low concomitant morbidity and a low incidence of respiratory events. A recent large-scale study published by van den Bunder et al. reported on preoperative apnea rates of about 5%. It is of particular interest to note that infants with preoperative respiratory abnormalities not only display marked alkalosis but also compensatory hypercapnia [9].

The present study examines the relationship between carbon dioxide and hemoglobin in children with hypertrophic pyloric stenosis. It demonstrates that these biomarkers are frequently underestimated at the time of diagnosis.

The authors are unaware of any published work that addresses the specific role of carbon dioxide in the pathophysiology of IHPS. Furthermore, the role of hemoglobin as a carbon dioxide transporter has yet to be the subject of a comprehensive investigation.

In general, central nervous respiratory drive and respiratory rate are decreased in metabolic alkalosis [10,11]. Advanced dehydration and alkalosis may lead to serious pre- and postoperative oxygen desaturation and apnea [9,12,13]. However, the pathophysiology of desaturation has not yet been clarified in detail. Due to the particular physiological importance of carbon dioxide, it can be expected that this volatile component of the acid/base balance plays a critical role.

Carbon dioxide is not only the “waste product” of oxidative metabolism. Rather, changes in the partial pressure of carbon dioxide (pCO_2_) affect a number of vital physiological functions, including the cerebral vascular tone and the blood flow. An increase in pCO_2_ in the blood may be due to either metabolic production or as a result of compensatory hypoventilation caused by alkalosis. With this in mind, it seems reasonable to look at hemoglobin as a transporter and intracellular buffer and lactate as a marker of hypoxia. In the literature, hemoglobin has hardly been considered as a biochemical marker in the context of IHPS. As far as we are aware, only one publication in the English-language literature specifically addresses the role of hemoglobin in infantile pyloric stenosis [14].

The hypercapnia that arises as a consequence of metabolic alkalosis exerts a range of effects on vital organs: the brain (inhibition of the respiratory drive, altered blood flow), the lungs (reduced alveolar gas exchange), and the kidneys (fluid, electrolyte and acid balance) are considered as effector organs in the context of acid/base balance.

The present study focuses on the pathophysiology and clinical significance of pCO_2_ and hemoglobin in IHPS. In particular, the interconnection between alkalosis, hypercapnia, hemoglobin and lactate will be analyzed.

## 2. Methods

### 2.1. Study Design

The data of 117 consecutive infants with IHPS that were treated between 2007 and 2010 and between 2014 and 2017 at the Department of Pediatric Surgery at the Ruhr University of Bochum, St. Marie’s Hospital, Herne, were retrospectively collected. Patient-related data were extracted from the personalized statutory German Yellow Book (particularly U1-Investigation at birth), the clinical records and the printouts of the acid/base analyzer. Diagnosis of IHPS was established by clinical appearance and ultrasound investigation and at laparoscopic pyloromyotomy. Twelve infants with incomplete core datasets were excluded from further analysis. The data of 105 infants were accepted for further data analysis. The following biometric and laboratory data were analyzed: birth weight (g), gestational age (postconceptional age at birth in weeks), postnatal age at admission (days), blood pH, carbon dioxide partial pressure (mmHg), bicarbonate (mmol/L), standard base excess (mmol/L), sodium, lactate (mmol/L), hemoglobin (g/dL) and hematocrit (%).

### 2.2. Study Framework

The primary aim of the study is to clarify the physiology of carbon dioxide partial pressure in relation to pathobiochemical changes in IHPS. In this context, particular attention was paid to the relationship with pCO_2_, which has been little investigated thus far and is of particular clinical interest. To explain these correlations, the following investigative steps were carried out.

1. First, we determined the respective mean and dispersion values for the investigated parameters (Table 1).
2. In the second step, we carried out corresponding correlation analyses focusing on the parameters pCO_2_, hemoglobin and lactate (Table 2). Additionally, we determined the quantitative dependence of the carbon dioxide partial pressure on standard bicarbonate and hemoglobin (linear regression analysis).
3. We used multivariate regression analysis to determine the main factors influencing the carbon dioxide partial pressure (Table 3).
4. A group comparison of biometric and biochemical parameters should reveal the differences between infants with elevated pCO_2_ values and those with normal pCO_2_ values (Table 4).
5. Finally, we investigated the constellations
  a. of preterm and term births (Table 5);
  b. of patients with low and normal hemoglobin levels (Table 6).

### 2.3. Clinical Pathway of Our Patients

All treatments were carried out in an inpatient setting. Initial routine blood tests usually involved taking a sample of capillary blood from the fingertip. This blood test was carried out as a point-of-care analysis. If IHPS was suspected clinically, depending on the symptoms, preoperative resuscitation was performed using a balanced, full-electrolyte intravenous solution with 5% glucose. A soft, diverting nasogastric tube sized 6–8 Charrière was inserted and removed immediately after pyloromyotomy. The infants underwent laparoscopic surgery the day after they were admitted to hospital. Feeding commenced four to six hours after the operation. During the preoperative and postoperative phases, the children were monitored clinically using pulse oximetry. On average, the acid/base status and ionogram were assessed twice or three times during the preoperative phase and once after the operation. Blood transfusions were not needed for any of the patients, nor was ventilation provided either before or after surgery. Depending on their tolerance of feeding, they were usually discharged home by the second or third day after the operation at the latest. The procedure was largely standardized, resulting in an average length of stay in hospital of 3 to 4 days. There were no unusual complications, and all of the children were discharged from hospital fully recovered.

### 2.4. Definitions and Stratification of Patients

The following inclusion criteria were selected: medical history of vomiting, ultrasound appearance of hypertrophic pylorus and postnatal age below 100 days. The exclusion criteria were as follows: vomiting caused by a different pathology; incomplete datasets; prior surgery for other conditions; and additional metabolic disturbances.

The gestational age corresponds to the calculated postconceptional age, which is measured in completed weeks. The age at presentation that we used corresponds to the uncorrected postnatal age in days.

Preterm infants were below 37 weeks of gestation. Full-term infants had a gestational age of at least 37 weeks of gestation.

As a cut-off point for normal or elevated pCO_2_, we used a partial pressure of 45 mmHg (6.0 kPa). We defined elevated pCO_2_ or hypercapnia as a pCO_2_ value exceeding 45 mmHg (6.0 kPa). Serious hypercapnia was assessed for values > 50 mmHg (6.6 kPa).

Anemia was defined according to the values of Zierk et al. (2019) [15].

**Figure 1:**
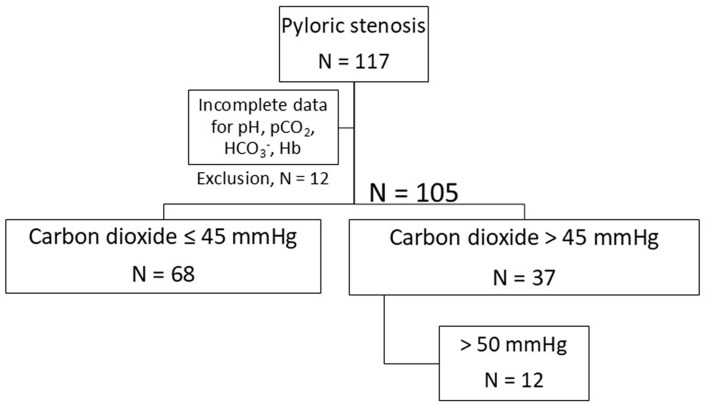
The stratification of patients according to their pCO_2_ levels.

In accordance with the main research question of the study, we performed a stratification according to the level of pCO_2_. Figure 1 provides an overview of the selection and categorization of our patients.

Furthermore, a comparison was conducted between the pCO_2_ and hemoglobin levels of premature babies up to 36 weeks of gestation and those of mature babies from 37 weeks of gestation. Mean corpuscular hemoglobin concentration (MCHC) was calculated as the ratio of hemoglobin concentration to hematocrit. Anemia was defined in accordance with the 10th percentile of age-related normal values, as proposed recently [15]. The lower limits of the normal range for infants aged less than 30 days are 10.6 g/dL; for those aged between 30 and 45 days, 9.4 g/dL; for those aged between 46 and 60 days, 9.4 g/dL; and for those aged 61 days and older, 9.6 g/dL. Based on the hemoglobin levels observed in our patients, we established a cut-off point of 10 g/dL for low hemoglobin.

### 2.5. Technical Considerations

Point-of-care analysis of capillary blood was routinely performed immediately after initial presentation on admission. The laboratory values therefore represent the infant’s condition before starting treatment. Acid/base and blood gas analyses were performed with a commercially available blood gas analyzer (GEM Premier 4000-Device, Instrumentation laboratory, Lexington, MA, USA). Blood samples were collected in heparinized capillary tubes and analyzed immediately.

### 2.6. Statistical Analysis

Data are presented as medians and interquartile ranges (IQRs). Means and standard deviations.

While the core datasets were fully available for analysis (*n* = 105), there were limitations in the dataset size for gestational age (*n* = 92), birth weight (*n* = 102), hematocrit (*n* = 98), MCHC (*n* = 98) and lactate (*n* = 80). The proportion of missing core data is 3.5%.

We used the statistical program Stata/IC 16.1 for Unix from StataCorp (4905 Lakeway Drive, College Station, TX, USA).

Generally, a *p*-value < 0.05 was considered statistically significant.

To test the normal distribution for each characteristic, we used the Shapiro–Wilk test. The assumption for the *t*-test is that the data is normally distributed. The *p*-value is shown in the column labeled “*p*-value #”. If this *p*-value is less than the significance level (0.05), then the null hypothesis of normally distributed data was rejected. If the *p*-value of the Shapiro–Wilk test was greater than 0.05, the group comparison was carried out using the Student’s *t*-test. The *p*-value in the *p*-value * column was then marked with a [t]. If the *p*-value of the Shapiro–Wilk test was less than 0.05, the group comparison was carried out using the Mann–Whitney U test. The *p*-value in the *p*-value * column was then marked with an [M].

We used violin plots to graphically represent our results. The plot shows the mean value (red circle) and the median (X). The slightly thicker line marks the 25% to 75% quartile (IQR) and the thinner lines going down and up are the so-called whiskers. The “bulbous” lines are an estimate of the distribution of the characteristic, so it is not the exact distribution that is shown but the so-called kernel density. As a result, the colored area for some characteristics goes beyond the actual range of values. You can see what the actual range of values is by looking at the outliers. If there is no outlier, then the minimum or maximum corresponds to the upper or lower whiskers. The small horizontal lines are the outliers.

Linear regression analysis was used to determine the relationship between the different parameters. The slope, intercept and Pearson’s correlation coefficient (r) were then determined. In order to identify the parameters most predictive of pCO_2_, multivariate linear regression analysis was applied.

To analyze the acid/base parameters of previous studies, we used the weighted mean, taking into account the respective sample sizes. For the comparison, we applied the arithmetic means of our study.

## 3. Results

### 3.1. Basic Data

**Figure 2:**
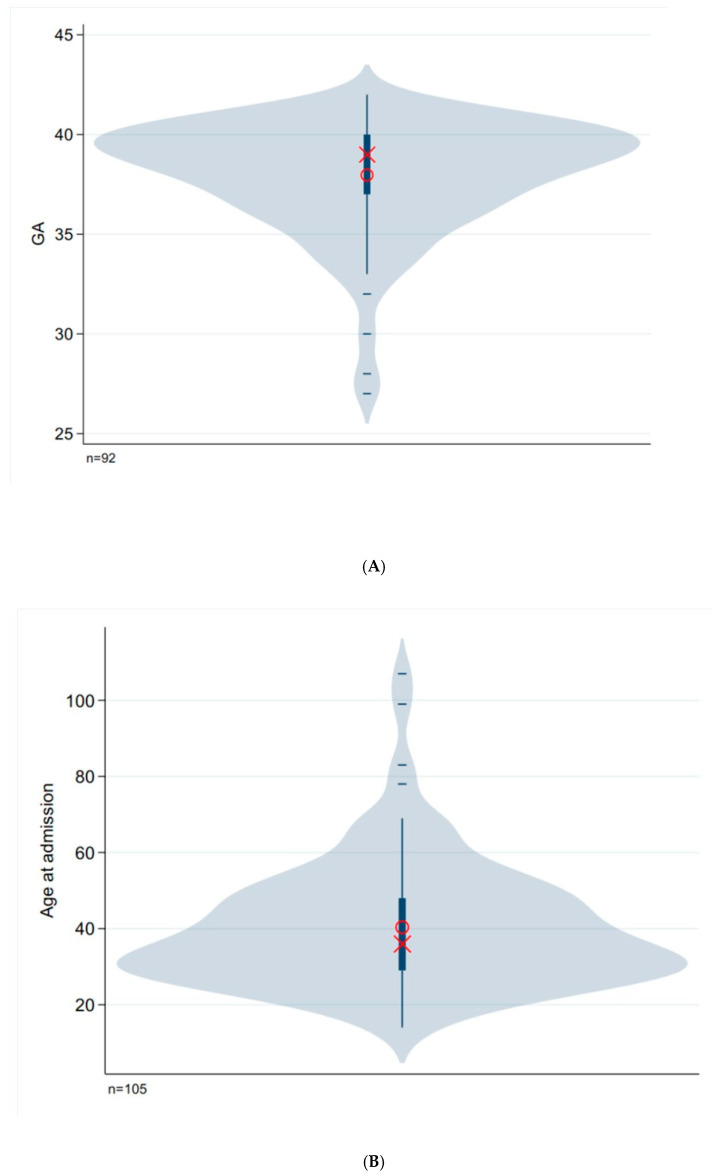

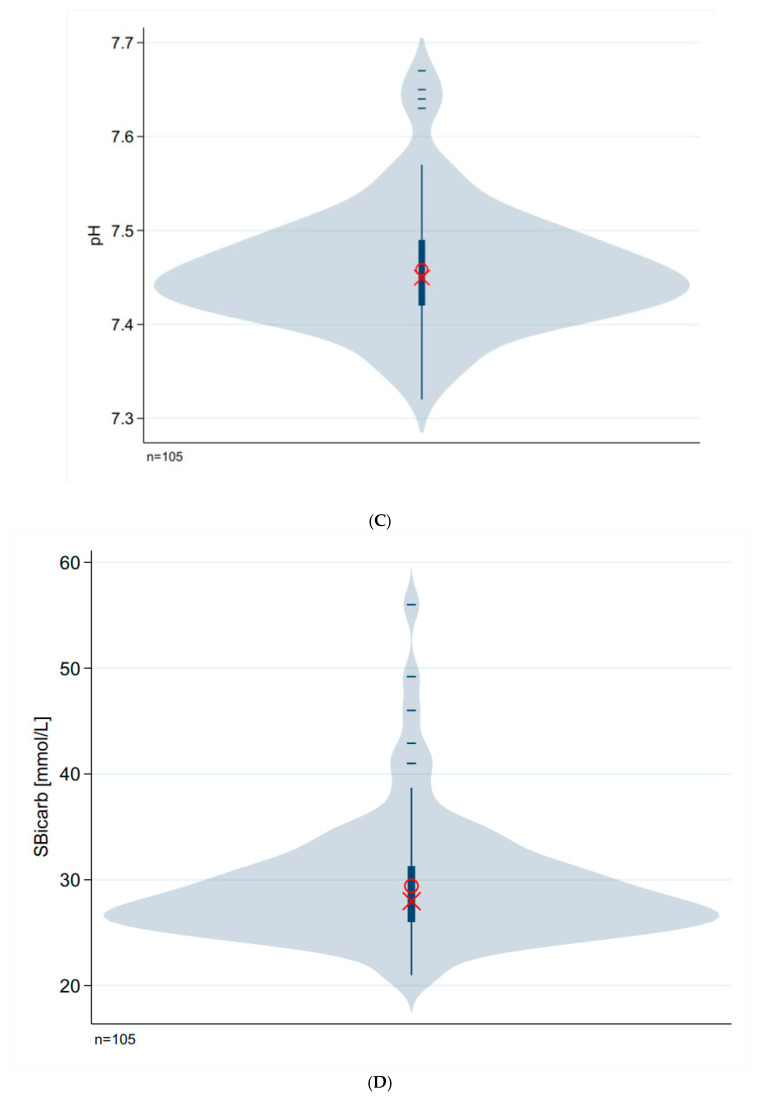

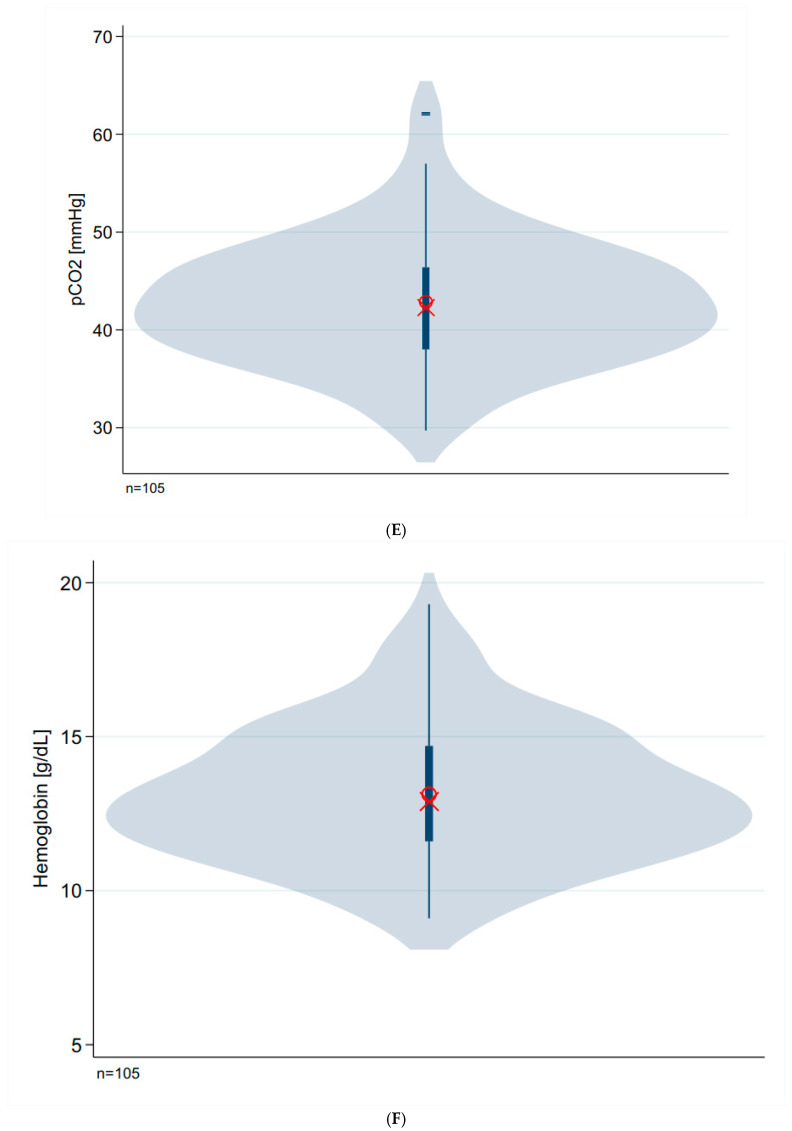
Graphical illustration of the value distributions for gestational age and age at presentation (**A**,**B**); the pH value (**C**); standard bicarbonate, (SBicarb, (**D**)); partial pressure of carbon dioxide (pCO_2_, (**E**)); and the hemoglobin concentration (**F**).

There was a male predominance in a ratio of 5:1. Median postconceptional/gestational age was 39 weeks [IQR 37–40] and postnatal age was 36 days (5.1 weeks) [IQR 29–48]. A total of 22 out of 92 children (equivalent to 23.9%) were born prematurely. Figure 2A,B illustrate gestational age and age at hospital admission, respectively. The graphs resemble a geometric mirror image of the ordinate axis of the coordinate system. It appears that premature infants develop IHPS later than full-term infants. At presentation, infants weighed 3850 g [IQR 3440–4300]. Table 1 presents the biometric, biochemical and acid/base status data. Ninety-three infants (89%) presented with normocapnia and 12 infants (11%) had hypercapnia > 50 mmHg pCO_2_. Only four children (4%) were anemic according to the data of Zierk et al. (2019) [15]. The mean values, scatter and different distribution patterns of key standard parameters and hemoglobin can be seen in Figure 2. Neither the pH value nor the standard bicarbonate concentration was normally distributed. The graphs show normal distributions for both carbon dioxide partial pressure and hemoglobin concentration. Both the pH value and standard bicarbonate show upward outliers (Figure 2C–F).

### 3.2. Correlation Analysis

**Figure 3:**
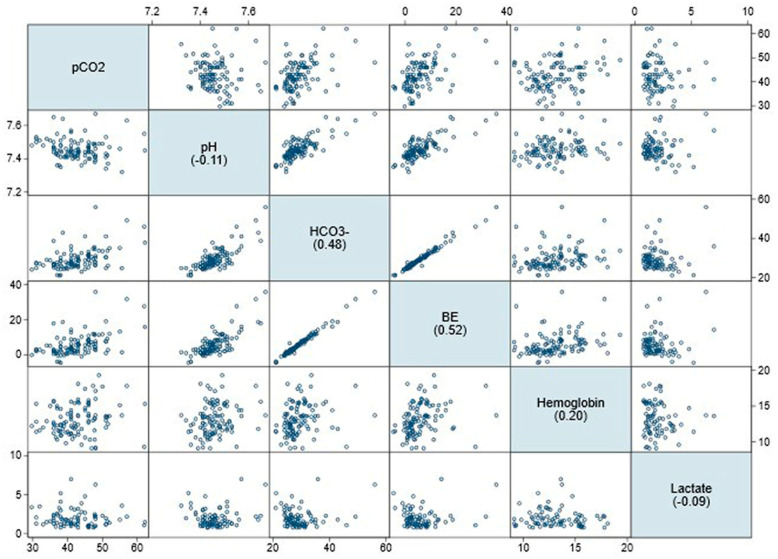
Scatterplot for the correlation of the laboratory results. The respective correlation coefficients for pCO_2_ are shown in the boxes with a blue background.

Correlation analysis revealed statistically significant positive correlations between SBicarb (r = 0.48, *p* < 0.001), pCO_2_ and base excess (r = 0.52, *p* < 0.01), as well as between pCO_2_ and hemoglobin (r = 0.20, *p* = 0.042). In addition, hemoglobin was positively correlated with the birth parameters (gestational age, r = 0.25, *p* = 0.016; birth weight (r = 0.3, *p* = 0.003). However, there was a negative correlation between hemoglobin and body weight at the time of admission (r = −0.3; *p* = 0.002). Furthermore, hemoglobin correlated positively with pH (r = 0.23, *p* = 0.020), SBicarb (r = 0.24, *p* = 0.013) and BE (r = 0.28, *p* = 0.004) (Table 2). The correlation between each of the parameters recorded is shown in a scatter plot in Figure 3. There was no significant link between pCO_2_ and demographic or biometric data (gestational age, birthweight, and body weight). Lactate was not correlated with either of the biometric or biochemical factors (Table 2 and Figure 3).

### 3.3. Linear Regression Analysis

Furthermore, we estimated the dependence of pCO_2_ on SBicarb, and hemoglobin.

pCO_2_ = 28.2 + 0.5 × [SBicarb], r = 0.48 (CI 0.32; 0.62), *p* < 0.001

The following regression equation relates pCO_2_ to hemoglobin concentration:

pCO_2_ = 35 + 0.6 × [Hemoglobin], r = 0.20 (CI 0.01; 0.38), *p* = 0.042

### 3.4. Identification of the Main Factors That Determine pCO_2_

Multivariate linear regression analysis showed that pCO_2_ was predominantly dependent on bicarbonate and base excess (both *p* < 0.001), pH (*p* < 0.001) and hemoglobin (*p* = 0.002) (Table 3).

### 3.5. Comparison of Infants with Normocapnia vs. Elevated pCO_2_ (Hyperkapnia)

The median pH values for the normocapnic and hypercapnic infants were both 7.5. Hemoglobin and hematocrit values were significantly higher in the high pCO_2_ group. We found significant differences in standard bicarbonate (*p* < 0.001), base excess (BE) (*p* < 0.001) and lactate (*p* = 0.005) (Table 4). There were no differences between the normocapnic and hypercapnic groups in terms of gestational age or birth weight. The median gestational age of the infants in the normocapnia group was almost the same (39 vs. 38 weeks, *p* = 0.128), and there was formally a higher birth weight in the hypercapnia group, though this was not significant.

**Figure 4:**
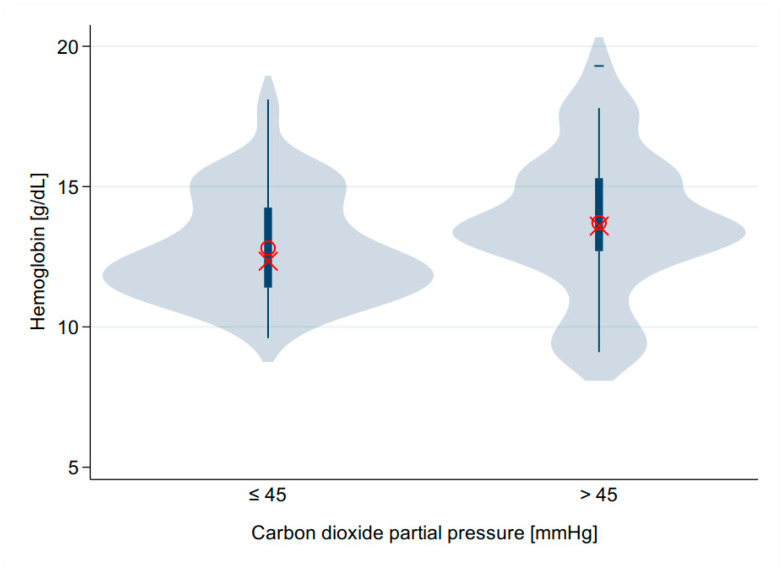
Violin plot of hemoglobin concentration. Comparison of the group with normocapnia ≤ 45 mmHg vs. group with pCO_2_ > 45 mmHg. The group with a pCO_2_ > 45 mmHg (6 kPa) had a higher hemoglobin concentration.

A graphical comparison of the hemoglobin concentrations of the groups with a normal or increased pCO_2_ reflects the significantly different distributions very well (Figure 4).

### 3.6. Comparison of pCO_2_ and Hemoglobin in Premature vs. Full-Term Infants

**Figure 5:**
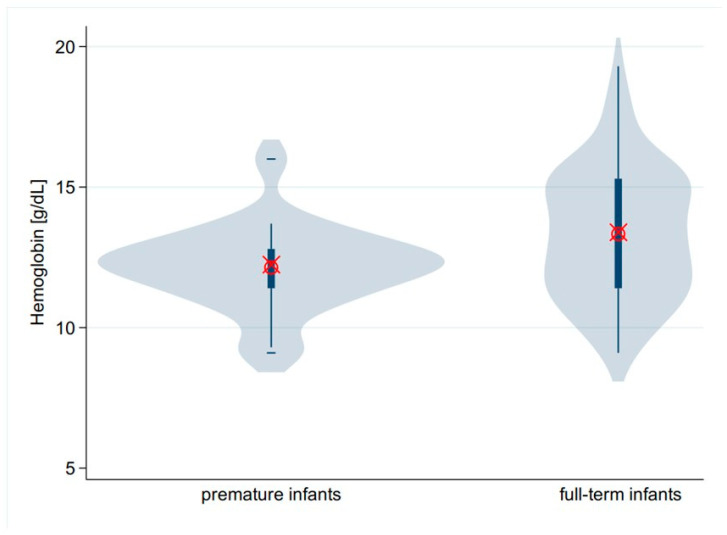
Preterm infants have lower hemoglobin levels than mature infants. However, the pCO_2_ is the same for both groups (see Table 5).

The comparison of the values for pCO_2_ as well as for hemoglobin and for MCHC of the premature infants with those of the mature infants in the cohort revealed a statistically significant difference for the hemoglobin concentration (12.2 vs. 13.4 g/dL, *p* = 0.006) (Table 5 and Figure 5). There was an increased postnatal age of premature babies at presentation (46 vs. 35 days, *p* = 0.074).

### 3.7. Comparison of Infants with Low Hemoglobin vs. Infants with Hemoglobin Above 10 g/dL

Only six infants with IHPS had a hemoglobin level below 10 g/dL (Table 6).

Children with a hemoglobin level below 10 g/dL had a lower gestational age (37 vs. 39 weeks, *p* = 0.048) and pCO_2_ values tended to be higher (47 vs. 42 mmHg, *p* = 0.135). These children also had a higher postnatal age (58 days vs. 36 days, *p* = 0.001). The mean corpuscular hemoglobin concentration (MCHC) was identical.

## 4. Discussion

The clinical picture of IHPS, often described as enigmatic, is characterized by a particularly distinctive pathophysiology. The results of the treatment are generally favorable. It is very likely to expect a normal quality of life in the long term.

**Figure 6:**
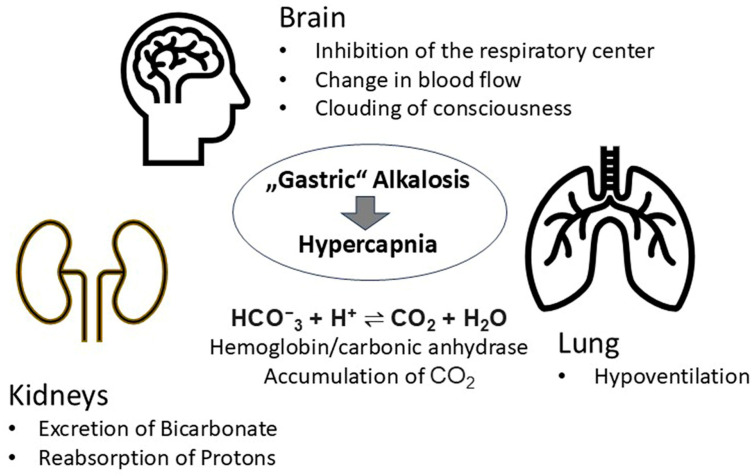
Regulation of the acid/base status in IHPS. Important effector organs are the lungs, kidneys and brain.

The repeated loss of gastric juice in IHPS results in the onset of a “gastric type” of metabolic alkalosis (Figure 6) [16]. An increase in pCO_2_ is the respiratory compensation for the metabolic changes. Accompanying respiratory disorders can become clinically relevant.

The potential danger of pre- and perioperative apnea has been questioned, but recent literature data have provided compelling evidence to confirm its existence [9,12,13,14,17,18,19]. The risk of a decreased respiratory drive in the preoperative period represents a potential clinical threat to the welfare of the child. Clinical signs of preoperative apnea may include shallow breathing, cyclical breathing or wheezing. Apnea may occur as a result of a central or obstructive cause [9].

The regulation of the acid/base status in IHPS is a subject that has been the focus of much research in recent years. It is imperative to note that the lungs, kidneys and brain are of particular significance in this regard (Figure 6).

Laboratory data for infants with IHPS are reported in a large number of publications. However, different weightings are applied with regard to the physiological significance [7,8,13,14,15,17,20,21,22,23,24].

### 4.1. Carbon Dioxide

Carbon dioxide is the major stimulus of respiratory drive and hemoglobin is a relevant transporter of the respiratory gases. Nevertheless, less than 5% of carbon dioxide is bound to hemoglobin as carbamate. However, this 5% contributes to 15% of the carbon dioxide gas exchange in the lungs. The remaining 80% of carbon dioxide in the blood is transported in the form of bicarbonate. Furthermore, carbon dioxide is bound as a physically dissolved gas in the plasma [25].

The action of hydrogen ions as a respiratory stimulant in the respiratory center of the brain stem has been demonstrated [26]. These are formed during the dissociation of carbonic acid into bicarbonate ions and hydrogen ions. Conversely, the absence of hydrogen ions in the context of metabolic alkalosis results in a reduction in respiratory drive, thereby causing hypoventilation and a secondary increase in carbon dioxide in the blood. This functions as a natural compensatory agent in the form of carbonic acid. The longer the history of pyloric stenosis, the more pronounced the alkalosis becomes. Furthermore, evidence indicates a positive correlation between the duration of the medical history and carbon dioxide levels [17]. As the disease progresses, an increase in pCO_2_ can be found. Feng et al. observed a markedly elevated pCO_2_ in the late onset group in comparison to the early onset group [27]. Nearly one-third of the children exhibited hypercapnia exceeding 45 mmHg (6 kPa) after a symptom duration more than ten days [27]. However, the carbon dioxide-associated compensation mechanism has natural limits. In the event of hypoxia due to hypoventilation, the lack of oxygen in the second instance takes over the trigger function for respiration [9]. It is unclear under what conditions this safety function, which is essential for survival, is also overridden.

### 4.2. Hypercapnia

In our study, we defined elevated carbon dioxide as a reading of 45 mmHg. This corresponds to 6.0 kPa. This threshold is consistent with previous studies [28]. The aim of this consideration was to better visualize changes in the potentially pathological range (Table 4 and Figure 4). Twelve infants (11.4%) had hypercapnia of more than 50 mmHg (>6.66 kPa, Figure 1).

The effects of carbon dioxide on consciousness and central nervous function must be considered in the context of elevated pCO_2_. In adults, an excess of bicarbonate above a threshold of 55 mmol/L may result in a notable elevation in pCO_2_. The regression equation of our data indicates that an SBicarb of 26 mmol/L (first quartile) is predictive of a pCO_2_ of 41 mmHg, while an SBicarb of 31 mmol/L (third quartile) is predictive of a pCO_2_ of 44 mmHg. Using the regression equation, we found that a bicarbonate of 55 mmol/L would result in a pCO_2_ of 56 mmHg. However, the bicarbonate dataset was not normally distributed, which significantly limits the reliability of a linear regression analysis.

This is likely due to the development of a coexisting respiratory muscle weakness resulting from severe hypokalemia, which is almost inevitable in this context [16]. Even moderate hypercapnia in the range of up to 70 mmHg leads to clouding of consciousness and respiratory inhibition. In particular, when pCO_2_ exceeds 90 to 100 mmHg in adults, this is known as carbon dioxide poisoning or acute hypercapnic respiratory failure [29,30]. Five infants in the present series exhibited pCO_2_ values between 55 and 62 mmHg. In these infants, an increased effect of hypercapnia on both alertness and respiratory drive would be expected. Furthermore, this range exceeds the upper limit of 52.5 mmHg (7 kPa), which has been proposed as a safe threshold for ventilated infants with permissive hypercapnia [28]. However, if IHPS is treated in time, the period of hypercapnia is short and long-term effects on the central nervous system are not of high concern.

### 4.3. Hemoglobin

New and, to the best of our knowledge and belief, probably for the first time in connection with an IHPS, is the proof of the interaction between hemoglobin and pCO_2_. We found a positive correlation between hemoglobin and pCO_2_ and a clearly increasing regression line slope (equal to 0.6 × hemoglobin). This finding reflects a general increase in pCO_2_ with increasing hemoglobin concentration.

The relationship of pCO_2_ with bicarbonate reflects the permanent chemical equilibrium between carbon dioxide and bicarbonate. According to the van Slyke equation, bicarbonate, hemoglobin and pH are the factors in the calculation of the artificial parameter base excess [31]. Despite the acidic effect of carbon dioxide, we found only no real tendency to lower pH in the hypercapnia group (Table 4). The regression analysis between pH and pCO_2_ showed a minimal slope of 0.001 (not significant). This tiny absolute value of the slope is of course due to the logarithmic nature of pH. When assessing the hemoglobin, the physiological trimester anemia of the former newborn must be taken into account. The vast majority of our patients were within the normal age/physiological range. In infants at postnatal age of six to nine weeks, fetal hemoglobin makes up more than half of the total hemoglobin. Under hypoxic conditions, fetal hemoglobin allows an improved oxygen delivery to the tissue compared to adult hemoglobin [32]. The data show that the age of predilection for IHPS overlaps with the developmentally predicted lowest hemoglobin level. In accordance with the consensus thresholds for operability, 41% (*n* = 43) of infants surpassed the ‘safety’ threshold for pCO_2_ of 43 mmHg (5.7 kPa) [33], yet no infant exhibited a critical hemoglobin level below 9 g/dl [33]. The occurrence of anemia appears to be rare, consistent with the limited existing literature on this subject [14,21,33,34]. It can be stated that anemia is an uncommon occurrence in the IHPS cohort. No child was below a threshold suggested by a recently published Delphi analysis [33]. Since both pCO_2_ and hemoglobin concentration are normally distributed, we performed a linear regression analysis of the two parameters. We found a positive correlation between hemoglobin and pCO_2_ and a clearly increasing regression line slope (equal to 0.6). This finding reflects a general increase in pCO_2_ with increasing hemoglobin concentration.

### 4.4. Hemoconcentration

In general, increasing length of vomiting in IHPS is associated with a trend towards sodium depletion [17,27]. Normo- or hyponatremia is usually found. In contrast, hypercapnia > 45 mmHg was not associated with increased sodium (Table 4). However, a significant increase in hematocrit was observed in the hypercapnia group. This is a reflection of hemoconcentration due to dehydration and alkalosis in the setting of hypercapnia. This observation is consistent with the rule that a contracted extracellular fluid volume can generally be assumed in gastric alkalosis [16]. Rehydration and administration of chloride and sodium ions as part of preoperative conditioning may reverse these changes and thus indirectly create the preconditions for normalization of carbon dioxide partial pressure. Conversely, although expansion of the extracellular fluid volume with sodium chloride dilutes blood bicarbonate to a small extent, its corrective effect in these patients is mainly a result of renal bicarbonate excretion [16].

### 4.5. Anemia

Infants with low hemoglobin had lower gestational age, higher pCO_2_ and higher postnatal age compared with the normal hemoglobin group (Table 6). Normally, the carbon dioxide balance is kept fairly constant under anemic conditions. In IHPS, the conditions are different. Alkalosis leads to an increase in the substrate for the carbonic anhydrase reaction in the form of increased bicarbonate. In addition, alkalosis and hydrogen ion deficiency lead to a slowing of respiration with reduced gas exchange. Physiological hyperventilation as a compensatory mechanism in anemia is therefore no longer possible. These mechanisms contribute to the establishment of an elevated equilibrium with increased pCO_2_ [25]. Camporesi et al. (2022) found a causal relationship between reduced postoperative hemoglobin concentration and the occurrence of postoperative apnea after narcosis and after pyloromyotomy [14]. According to this study, postoperative apnea affects about 1 in 10 children with IHPS. Furthermore, these authors draw the conclusion that anemia is associated with reduced oxygen transport capacity and reduced oxygen supply to the brain [14]. This in turn would reduce the efferent output of the respiratory center. Significant evidence for an impaired cerebral and renal oxygenation under conditions of marked alkalosis has been reported in a previous study by our research group [35].

In contrast to the findings of Camporesi et al. (2022) [14], a large-scale epidemiological investigation demonstrated that preoperative anemia has no impact on the postoperative outcome following pyloromyotomy. This study encompassed infants under one year of age, with anemia defined as a hematocrit of less than 40% irrespective of age [34].

In infants with metabolic alkalosis, it is also necessary to consider the effects on the oxygen binding curve of hemoglobin. Alkalosis results in a stronger binding of oxygen to hemoglobin, which in turn leads to a reduction in the effective release of oxygen to the tissue (Bohr effect). This effect can be reversed by normalizing the pH value towards normal. Previously, we were able to demonstrate that cerebral oxygenation can be normalized in the context of preoperative fluid therapy and conditioning [35].

### 4.6. Lactate

It is commonly assumed that an elevation in lactate levels is indicative of a negative outcome. However, this assumption is not entirely accurate. In addition to its role in facilitating brain metabolism, lactate has been demonstrated to enhance cognitive function [36,37]. However, lactate plays a minor role in the routine diagnostic workup of IHPS. There is almost no specific information on the pathophysiological role of lactate in children with IHPS. When the lactate levels of babies with IHPS were compared with babies who vomited for other reasons, there were no differences [17]. Infants with IHPS did not differ in lactate concentration at hospital admission, pre- or postoperatively [14].

Surprisingly, infants with hypercapnia formally had a lower median lactate compared with normocapnoic infants (Table 4). While this difference is not statistically significant, it may nevertheless be indicative of a genuine trend. In this context, it is necessary to consider the potential influence of hypercapnia and alkalosis on glycolysis and gluconeogenesis, given the complex interaction between these factors. Conversely, it is possible that an erroneous conclusion regarding elevated lactate values may be derived from the analysis of some individual venous blood samples. It seems that an increase in pCO_2_ was not associated with an increase in anaerobic glycolysis, as measured by lactate levels. The influence of hypercapnia in metabolic alkalosis on lactate requires further clarification.

### 4.7. Preterms

In alignment with the findings presented in Table 5, prior research has demonstrated that preterm infants with IHPS tend to manifest symptoms at a later stage. Their clinical course is more complex, and the length of hospitalization is typically longer [38,39,40]. In our study, which has a high proportion of preterm infants, this is reflected by the fact that pyloric stenosis tends to have a relatively late onset (in terms of age at admission). Our patients have a higher postnatal age compared to the results of the comparative studies. The inverse-identical distribution pattern of gestational age in comparison to age at hospital admission is indicative of this unique characteristic of preterm infants with IHPS (Figure 2A,B). Our results indicate that preterm infants did not have lower hemoglobin or higher pCO_2_ compared with term infants (Table 5). It is probable that reduced respiratory drive or pulmonary dysfunction had played a subordinate role in this preterm subgroup.

### 4.8. Clinical Considerations

For clinical practice, we recommend blood gas analysis and pulse oximetry monitoring of each child at presentation with IHPS and dehydration. Nevertheless, there is a need for further research to establish generally accepted guidelines for preoperative monitoring, which should also take prematurity and hemoglobin into account. The use of near-infrared spectroscopy (NIRS) in a clinical setting or scientific context has the potential to yield more comprehensive perioperative data regarding the oxygenation of the brain and other organs [35].

In the event of respiratory failure and hypoxia, respiratory supplementation may be required. It should be noted that oxygen administration is expected to displace hemoglobin-bound CO_2_ (Haldane effect) [25]. Such an intervention could potentially exacerbate central respiratory depression, thereby inducing apnea as a consequence of iatrogenic factors.

Over the last three decades, laparoscopic pyloromyotomy has increasingly become the standard method internationally, with its popularity growing steadily [41]. This is particularly true of high-income countries. The data presented in our series relate to infants who underwent laparoscopic surgery using the three-trocar technique. The first report on laparoscopic pyloromyotomy was by Alain, Grousseau and Terrier from Limoges (France) in 1990 [42].

### 4.9. Limitations

A significant limitation of our study is its retrospective and single-institution design. This has resulted in a relatively small number of patients being included in the study. Furthermore, the absence of clinical data on the preoperative course precludes a direct clinical correlation. As the study was retrospective in nature, only a reduced dataset was available for statistical evaluation of certain facts. All data were obtained from routine blood samples. It can be reasonably assumed that the measured values of the specific soluble and volatile parameters are influenced by a number of factors, including the sampling conditions, such as the location from which the sample is taken, the length of time the sample has been stored, and the temperature. In routine practice, the sample used was typically capillary blood from the non-arterialized fingertip. However, in certain instances, venous blood may also have been used for the determination of the aforementioned parameters. It has been shown that the results of acid/base and blood gas analyses in newborns correlate with each other and are largely interchangeable [43]. Even storage times of 1 to 2 h, as could possibly occur in cases of acceptance, appear to have only a minor effect on the results [44]. As blood sampling is associated with a painful stimulus, babies usually react by crying (hyperventilation) and sometimes also with brief respiratory arrest (hypoventilation). This leads to rapid changes in blood gases, which are detectable in arterial and therefore capillary blood samples, but less so in venous blood samples. Capillary blood samples may therefore reflect a short-term condition. Conversely, venous blood would be better suited to reflect the longer-term basic condition [45]. The analyzer we used is subject to continuous quality control, which meets the legal requirements. It is important to note that the values obtained via a point-of-care analyzer cannot be directly equated with those of an automatic laboratory analyzer [46]. This is particularly relevant in the context of hemoglobin concentration and hematocrit. It is also notable that higher hemoglobin and hematocrit values are typically observed in capillary blood compared to venous blood [47].

## 5. Conclusions

The pathophysiology of carbon dioxide in IHPS requires special consideration. Due to the direct effect of carbon dioxide on the central nervous system, it is likely to play a particularly important role in the development of preoperative apnea. The findings of our study demonstrated that infants diagnosed with IHPS comprise a subgroup of children exhibiting elevated partial pressures of carbon dioxide in the blood, thereby highlighting a correlation with respiratory physiology. The role of hemoglobin in the pathophysiology of IHPS remains controversial. Our findings suggest that increased transport capacity by hemoglobin is associated with increased pCO_2_. Therefore, an increase in carbon dioxide cannot easily be attributed to reduced carbon dioxide transport function due to hemoglobin deficiency. To gain a more nuanced understanding of these intricate connections, conducting an investigation using Stewart’s physico-chemical model might be an option, as it will yield valuable in-depth insights.

## Figures and Tables

**Table 1:** Demographic data and laboratory results. Shapiro–Wilk test for normality.

Parameter	*N*	Median (IQR)	Mean (SD)	*p*-Value #
Gestational age [weeks]	92	39.0 (3.0)	38.0 (2.8)	<0.001
Age at presentation [d]	105	36.0 (19)	40.4 (16.4)	<0.001
Birth weight [g]	102	3215 (840)	3213 (681)	0.004
Weight at presentation [g]	105	3850 (860)	3917 (740)	0.245
pH	105	7.5 (0.1)	7.5 (0.1)	<0.001
pCO_2_ [mmHg]	105	42.0 (8.0)	42.9 (6.2)	0.087
SBicarb, HCO_3_^−^ [mmol/L]	105	28.0 (5.3)	29.4 (5.5)	<0.001
BE [mmol/L]	105	4.6 (6.4)	6.1 (6.5)	<0.001
Sodium [mmol/L]	105	137.0 (3.0)	136.9 (3.2)	<0.001
Lactate [mmol/L]	80	1.8 (1.2)	2.0 (1.2)	<0.001
Hemoglobin [g/dL]	105	12.9 (3.1)	13.1 (2.2)	0.080
Hematocrit	98	36.5 (10.0)	37.9 (7.7)	0.001
MCHC [g/dL]	98	35.1 (2.7)	35 (2.6)	0.001

Abbreviations: #, Shapiro–Wilk test for normality; IQR, interquartile range; pCO_2_, carbon dioxide partial pressure; SBicarb, standard bicarbonate; BE, base excess; MCHC, mean corpuscular hemoglobin concentration.

**Table 2:** Correlations of carbon dioxide partial pressure and hemoglobin concentration with single parameters. In addition, data on lactate is provided.

Parameter	*N*	rho (95%-CI)	*p*-Value
Carbon dioxide partial pressure (pCO_2_)			
Gestational age [weeks]	92	−0.12 (−0.32; 0.09)	0.250
Birth weight [g]	102	0.03 (−0.17; 0.22)	0.783
Weight at admission [g]	105	−0.10 (−0.29; 0.09)	0.290
pH	105	−0.11 (−0.30; 0.08)	0.262
SBicarb, HCO_3_^−^ [mmol/L]	105	0.48 (0.32; 0.62)	<0.001
BE [mmol/L]	105	0.52 (0.36; 0.65)	<0.001
Hemoglobin [g/dL]	105	0.20 (0.01; 0.38)	0.042
MCHC [g/dL]	98	−0.13 (−0.32; 0.07)	0.214
Lactate [mmol/L]	80	−0.09 (−0.30; 0.14)	0.443
Hemoglobin			
Gestational age [weeks]	92	0.25 (0.05; 0.43)	0.016
Birth weight [g]	102	0.30 (0.11; 0.46)	0.003
Weight at admission [g]	105	−0.30 (−0.46; −0.11)	0.002
pH	105	0.23 (0.04; 0.40)	0.020
SBicarb, HCO_3_^−^ [mmol/L]	105	0.24 (0.05; 0.41)	0.013
BE [mmol/L]	105	0.28 (0.09; 0.44)	0.004
MCHC [g/dL]	98	−0.18 (−0.37; 0.02)	0.073
Lactate [mmol/L]	80	−0.07 (−0.29; 0.15)	0.520
Lactate			
Gestational age [weeks]	73	−0.01 (−0.24; 0.22)	0.908
Birth weight [g]	79	−0.06 (−0.28; 0.16)	0.570
Weight at admission [g]	80	0.06 (−0.16; 0.28)	0.585
pH	80	0.10 (−0.12; 0.31)	0.382
SBicarb, HCO_3_^−^ [mmol/L]	80	0.10 (−0.12; 0.31)	0.368
BE [mmol/L]	80	0.10 (−0.12; 0.31)	0.369
MCHC [g/dL]	75	−0.13 (−0.34; 0.10)	0.279

Abbreviations: pCO_2_, carbon dioxide partial pressure; SBicarb, standard bicarbonate; BE, base excess; MCHC, mean corpuscular hemoglobin concentration; r (rho), Pearson’s correlation coefficient; CI, confidence interval.

**Table 3:** The results of multivariate linear regression analysis.

	Full Model		Stepwise with SBicarb	
	Coeff (95%-CI)	*p*-Value	Coeff. (95%-CI)	*p*-Value
Weight	0.20 (0.25; 0.64)	0.388	†	†
pH	−125.7 (−134.3; −117.1)	<0.001	−126.9 (−138.4; −115.3)	<0.001
SBicarb	−0.21 (−0.86; 0.43)	0.511	1.6 (1.4; 1.9)	<0.001
Base excess	1.6 (1.1; 2.0)	<0.001	N.a.	N.a.
Hemoglobin	0.22 (0.08; 0.37)	0.003	0.39 (0.15; 0.63)	0.002
Constant	973.0 (913.1; 1033.0)	<0.001	936.4 (854.0; 1018.9)	<0.001
AIC	425.0	†	479.0	†

Abbreviations: AIC, Akaike Information Criteria; CI, confidence interval; SBicarb, standard bicarbonate; †, not sensible.

**Table 4:** Comparison of the normocapnia ≤ 45 mmHg vs. group with pCO_2_ > 45 mmHg. Median value (IQR).

Parameter	Normal pCO_2_ ≤ 45 mmHg	N	*p*-Value Normal pCO_2_ #	pCO_2_ > 45 mm Hypercapnia Hy	N	*p*-Value Elevated pCO_2_ #	*p*-Value *
Gestational age [weeks]	39 (3)	64	< 0.001	38 (2.5)	28	0.001	0.128 [M]
Birthweight [g]	3162 (1010)	68	0.069	3315 (690)	34	0.010	0.678 [M]
pH	7.5 (0.1)	68	0.009	7.5 (0.1)	37	0.017	0.561 [M]
pCO_2_ [mmHg]	39.8 (5.0)	68	†	48 (5.0)	37	†	†
SBicarb, HCO_3_^−^ [mmol/L]	27.0 (3.7)	68	<0.001	31.1 (5.3)	37	<0.001	<0.001 [M]
BE [mmol/L]	3.0 (4.6)	68	<0.001	8.1 (5.9)	37	<0.001	<0.001 [M]
Sodium [mmol/L]	137 (2.5)	68	0.320	137 (3.0)	37	0.001	0.628 [M]
Lactate [mmol/L]	1.8 (1.4)	54	<0.001	1.4 (1.2)	26	<0.001	0.025 [M]
Hemoglobin [g/dL]	12.4 (2.8)	68	0.001	13.6 (2,6)	37	0.198	0.026 [M]
Hematocrit	35.6 (8.0)	65	0.001	40.0 (8.7)	33	0.432	0.031 [M]
MCHC [g/dL]	35.5 (2.9)	65	0.020	34.6 (2.1)	33	0.003	0.077 [M]

Abbreviations: IQR, interquartile range; pCO_2_, carbon dioxide partial pressure; SBicarb, standard bicarbonate; BE, base excess; MCHC, mean corpuscular hemoglobin concentration; †, not sensible; #, Shapiro–Wilk test for normality; * *p* value: [M] Mann–Whitney U test.

**Table 5:** Comparison of the values for pCO_2_, hemoglobin and MCHC of twenty-two preterm infants with those of full-term infants. Median, IQR.

	Preterm	*N*	*p*-Value Preterm #	Full Term	*N*	*p*-Value Term #	*p*-Value *
Gestational age [weeks]	35.0 (2.8)	22	<0.001	39 (2.0)	70	0.120	†
Age at presentation [d]	46.0 (25.5)	22	0.976	35.0 (17)	70	<0.001	0.074 [M]
pCO_2_ [mmHg]	41.9 (8.4)	22	0.009	41.7 (8.4)	70	0.271	0.769 [M]
Hemoglobin [g/dL]	12.2 (1.4)	22	0.308	13.4 (3.9)	70	0.222	0.006 [t]
MCHC [g/dL]	34.4 (3.8)	22	0.136	35.4 (2.8)	66	<0.001	0.673 [M]

Abbreviations: IQR, interquartile range; pCO_2_, carbon dioxide partial pressure; MCHC, mean corpuscular hemoglobin concentration; †, not applicable; #, Shapiro–Wilk test for normality; * *p* value: [M] Mann–Whitney U test, [t] two-sample *t* test.

**Table 6:** Comparison of the values for pCO_2_, hemoglobin and MCHC of infants with hemoglobin < 10 g/dl with those of infants with hemoglobin ≥ 10 g/dL. Median value (IQR).

	Low Hb	*N*	*p*-Value, Low Hb #	Normal Hb	*N*	*p*-Value, Low Hb #	*p*-Value *
Gestational age [weeks]	37 (4)	6	0.326	39 (3.0)	86	<0.001	0.044 [M]
Age at presentation [d]	58 (12)	6	0.413	36 (18)	99	<0.001	0.001 [M]
pCO_2_ [mmHg]	47.5 (4)	6	0.269	42 (18)	99	0.376	0.135 [t]
Hemoglobin [g/dL]	9.2 (0.5)	6	0.210	13.0 (3.2)	99	0.001	†
MCHC [g/dL]	34.7 (4)	6	0.741	35.2 (2.6)	92	<0.001	0.932 [M]

Abbreviations: IQR, interquartile range; Hb, hemoglobin; pCO_2_, carbon dioxide partial pressure; MCHC, mean corpuscular hemoglobin concentration; †, not applicable; #, Shapiro–Wilk test for normality; * *p* value: [M] Mann–Whitney U test, [t] two-sample *t* test.

## Data Availability

Data openly available in a public repository that issues datasets at https://doi.org/10.17605/OSF.IO/KJQFP.
